# Mechanism Analysis of Bubble Discharge Within Silicone Gels Under Pulsed Electric Field

**DOI:** 10.3390/gels10120799

**Published:** 2024-12-06

**Authors:** Dongxin He, Zhe Zhang, Guangzhu Wang, Keming Liu, Haochen Wang, Zhe Xu, Gilbert Teyssedre, Yuantao Zhang

**Affiliations:** 1 School of Electrical Engineering, Shandong University, Jingshi Road 17923, Jinan 250061, China; hdx@sdu.edu.cn (D.H.); 202314676@mail.sdu.edu.cn (Z.Z.); 202234738@mail.sdu.edu.cn (H.W.); xuzhebm@163.com (Z.X.); 2 Shandong Provincial Key Lab of UHV Technology and Modern Power Electronics Technology and Its Applications, School of Electrical Engineering, Shandong University, Jinan 250061, China; sdwgz@sdu.edu.cn; 3 Nari Semiconductor Co., Ltd., Nanjing 210003, China; liukeming@sgepri.sgcc.com.cn; 4 Laboratoire Plasma et Conversion d’ Energie, University of Toulouse, 31000 Toulouse, France; gilbert.teyssedre@laplace.univ-tlse.fr

**Keywords:** pulsed electric field, silicone gels, electrical treeing, bubble discharge

## Abstract

Silicone gel, used in the packaging of high-voltage, high-power semiconductor devices, generates bubbles during the packaging process, which accelerates the degradation of its insulation properties. This paper establishes a testing platform for electrical treeing in silicone gel under pulsed electric fields, investigating the effect of pulse voltage amplitude on bubble development and studying the initiation and growth of electrical treeing in a silicone gel with different pulse edge times. The relationship between bubbles and electrical treeing in silicone gel materials is discussed. A two-dimensional plasma simulation model for bubble discharge in silicone gel under pulsed electric fields is developed, analyzing the internal electric field distortion caused by the response times of different ions and electrons. Additionally, the discharge current and its effects on silicone gel under pulsed electric fields are examined. By studying the influence of different pulse edge times, repetition frequencies, and temperatures on discharge current magnitude and ozone generation rates, the impact of electrical breakdown and chemical corrosion on the degradation of organic silicone gel under various operating conditions is analyzed. This study explores the macroscopic and microscopic mechanisms of dielectric performance degradation in organic silicone gel under pulsed electric fields, providing a basis for research on high-performance packaging materials and the development of high-voltage, high-power semiconductor devices.

## 1. Introduction

The insulated-gate bipolar transistor (IGBT), as a core component of energy conversion and transmission system, offers numerous advantages, such as energy-saving, high efficiency, environmental friendliness, and ease of installation, making it widely used in various applications [1,2]. Silicone gels are commonly used in the packaging of IGBT devices due to their high elasticity, low stress, and good insulation properties, which effectively protect the internal structure of power semiconductors [3,4].

With the continuous miniaturization of electronic devices and the increase in operating frequencies, the tiny bubbles present in the material due to the manufacturing process have a significant impact on the insulation properties of these modules during operation [5,6]. Extensive research has been conducted on the effects of bubbles on the electrical treeing characteristics of silicone gels [7,8,9]. Some scholars have studied the partial discharge characteristics of the DBC–silicone gel structure in IGBT modules under square-wave pulses and have conducted theoretical analyses on the impact of small bubbles. They found that bubble defects more easily induce discharge, leading to the degradation of the material’s electrical properties [10,11]. It is clear that bubbles inside a silicone gel are a key factor influencing its insulation properties. However, the existing studies have only explored the electrical treeing and breakdown failure mechanisms of silicone gels under conventional electric fields, and the specific degradation mechanisms under pulsed electric fields remain unclear.

A large number of studies on the local discharge characteristics of silicone gels have generally found bubbles at the tips of electrical trees and have explored the relationship between the bubbles and the growth of electrical treeing [12,13]. However, most studies have only reported the bubble characteristics after the formation of electrical trees, with limited research on how the bubbles in the silicone gel’s crosslinked network evolve in response to electric field changes and how they induce electrical treeing before the formation of electrical trees.

Compared with direct current (DC) and alternating current (AC) electric fields, the rapid change in amplitude at the edges of pulsed electric fields is a notable feature, which may be closely related to the failure and degradation of silicone gel. Scholars both domestically and internationally have conducted extensive experiments to investigate the effect of a pulse field’s edge time on insulation degradation. For example, You Haoyang studied local discharge behavior at the triple junction of power devices encapsulated with silicone gel and found that the initiation voltage of local discharge decreased linearly with the decrease in the square-wave pulse field’s edge time, while the attenuation rate of local discharge increased with the decrease in the width of the square-wave pulse field [10]. However, most current research focuses on the impact of pulse edge time on local discharge, with limited studies on the effects of pulse edge time on the characteristics of electrical treeing within the dielectric.

Experimental research on bubble discharge under pulsed electric fields cannot visualize the internal changes during discharge. Therefore, numerical simulations are needed to better describe the generation and migration of positive and negative charges under the pulsed electric field and to explore the microscopic mechanisms of bubble discharge [14,15,16,17]. Current bubble discharge simulation models are mainly divided into two types: the three-capacitor model and the finite element simulation model. The three-capacitor circuit model simplifies the bubble-containing solid insulation material into a capacitive network, which can only show the transient changes in charge at the electrode ends, with little involvement in the charge process itself [18]. Therefore, to more accurately describe the discharge process, a finite element discharge simulation model has been proposed. Most current studies focus on the current characteristics and charge behavior of partial discharge processes in air gaps under power frequency AC electric fields, while a few studies on pulsed electric fields have not analyzed the chemical reactions occurring during discharge or the effects of discharge by-products on the material [18,19].

Relevant studies have shown that the ozone molecules formed during discharge are an important indicator in material degradation research [20]. Ozone, as a strong oxidizing agent, can trigger the chemical degradation and surface oxidation of the silicone matrix under UV conditions, thereby accelerating the performance degradation of insulation materials and also affecting their dielectric properties [21]. Therefore, exploring the generation and development of ozone molecules under pulsed electric fields not only helps reveal the microscopic mechanisms of bubble discharge but also provides guidance in terms of improving the durability of silicone gels.

To investigate the relationship between bubbles at the needle tip and the initiation of electrical treeing under pulsed electric fields, this study uses WACKER’s next-generation, high-temperature-resistant, high-insulation addition-curing silicone gel and establishes an electrical treeing testing platform [22]. The morphology and development process of bubbles within the silicone gel under pulsed electric fields were preliminarily studied, and the influence of different edge times and voltage amplitudes on electrical treeing development was tested. Additionally, to further explore the evolution process of bubble discharge under pulsed electric fields and its impact on material insulation, this study considers the chemical reactions involved in air–medium discharge and develops a simulation model suitable for bubbles within a silicone gel under pulsed electric fields. The model quantitatively analyzes the spatiotemporal evolution process of bubble discharge and explores the mechanism of its impact on the insulation degradation of silicone gel. Furthermore, the effects of different high-voltage IGBT operating conditions (such as varying pulse edge times, repetition frequencies, and temperatures) on bubble discharge are discussed. These findings provide a theoretical foundation for improving the insulation performance of silicone gels and advancing the development of packaging materials for device insulation.

## 2. Results and Discussion

### 2.1. Bubble Characteristics of Silicone Gel Under Pulsed Electric Field

**Figure 1:**
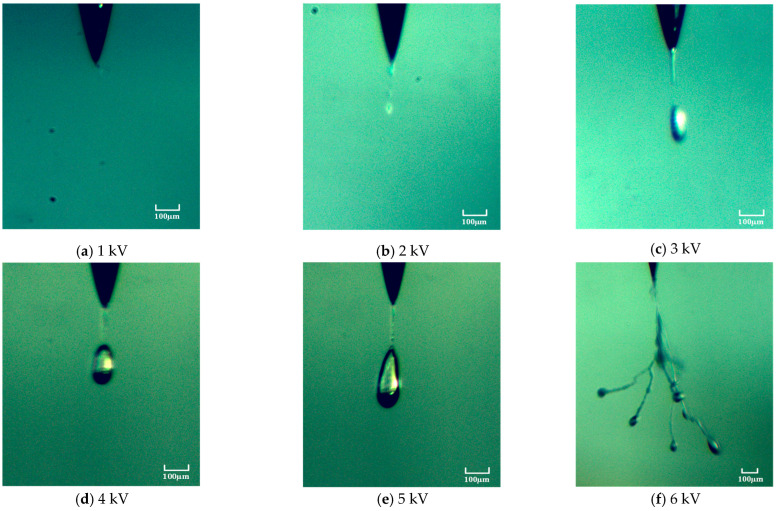
The variation law of bubble shape near the needle tip with the amplitude of pulse voltage.

To clarify the bubble generation mechanism, the variation in bubble morphology near the needle tip with respect to the voltage amplitude was investigated, as shown in Figure 1. When the square-wave pulsed voltage amplitude exceeds 2 kV, bubbles begin to form near the needle tip. As the square-wave pulsed voltage amplitude increases, the bubble volume gradually increases, and the distance between the bubble and the needle tip also lengthens. The bubble morphology changes from ellipsoidal to droplet-like, as shown in Figure 1c–e. When the square-wave pulsed voltage amplitude is greater than or equal to 6 kV, no bubbles are observed near the needle tip; instead, electrical treeing occurs directly, as shown in Figure 1f. The results indicate that the pulsed voltage amplitude has a significant impact on the bubble morphology.

**Figure 2:**
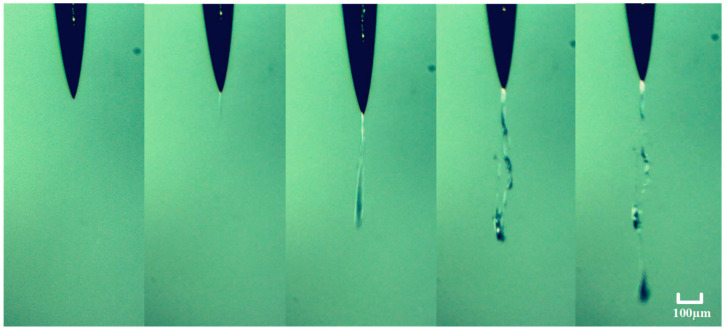
Dynamic process of electrical tree generation in silicone gel.

The dynamic process of electrical tree formation at the needle tip is shown in Figure 2. As seen in the figure, a white, filament-like channel first forms at the needle tip, followed by the appearance of a narrow bubble at the end of the channel. The bubble rapidly grows and induces cracks in the surrounding medium, eventually forming an electrical tree. The entire process, from the formation of the fine filament-like channel to the development of the electrical tree, occurs very quickly, in just 0.17 s.

**Figure 3:**
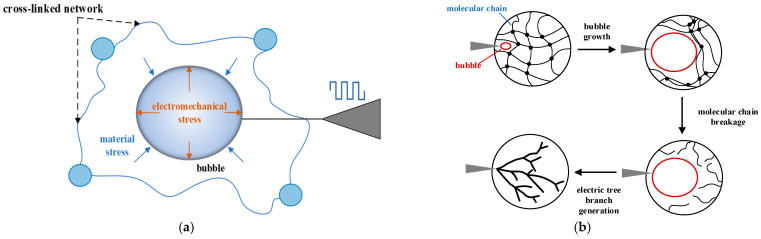
(**a**) A schematic diagram of stress on bubbles in silicone gel; (**b**) a schematic diagram of bubble expansion inducing the internal electrical treeing process in silicone gel.

In addition to the electro-mechanical stress induced by the electric field, the bubble also experiences material stress exerted by the crosslinked network, which acts to resist the expansion of the bubble’s volume. The force diagram of the bubble is shown in Figure 3a. The pulsed electric field causes the air gap to expand, and the volume expansion of the bubble leads to an instantaneous compression of the silicone gel’s crosslinked network, resulting in a dramatic increase in the internal stress experienced by the molecular chains due to the volume change.

When the electric field intensity is low, the bubble expansion is small, and the stress generated in the surrounding molecular chains is insufficient to cause chain rupture. Therefore, only bubbles are observed near the needle tip, and no electrical treeing occurs. However, at higher electric field intensities, the high frequency of the pulsed electric field causes the bubble volume to expand sharply when the electric field force exceeds the material stress. The surrounding molecular chains break under the stress, forming cracks, which further develop under the influence of the electric field, leading to the formation of electrical treeing, as shown in Figure 3b.

Experimental results indicate that, in most cases, electrical treeing in silicone gel originates from bubble development. The rapid expansion of the bubble under the influence of the electric field and the internal chemical processes are the direct triggers for the formation of electrical treeing. Additionally, bubbles near the needle tip can only be observed under the condition of a square-wave pulse superimposed on a DC electric field, suggesting that the expansion of the bubble is heavily influenced by electro-mechanical stress.

### 2.2. The Influence of Pulsed Electric Field Edge Time on the Development of Electrical Treeing in Silicone Gel

**Figure 4:**
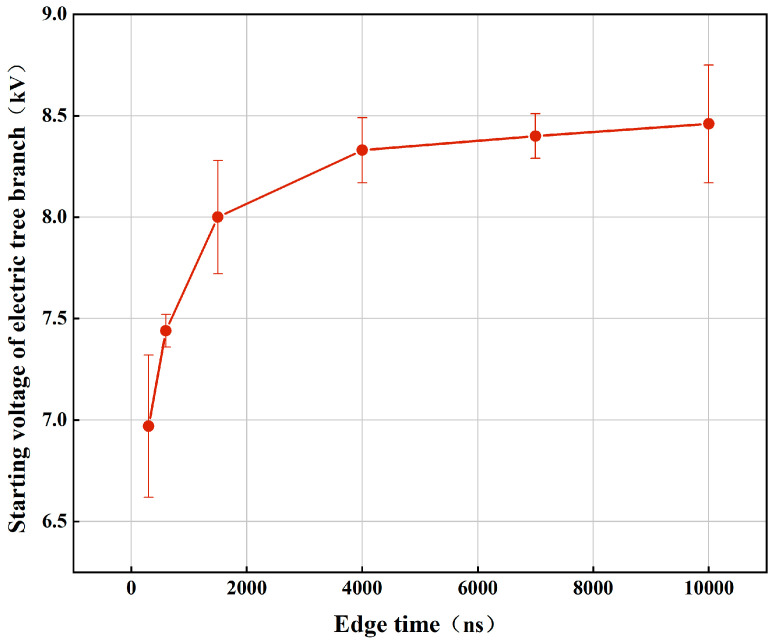
The variation pattern of electrical treeing inception voltage in silicone gel with pulsed electric field edge time.

**Figure 5:**
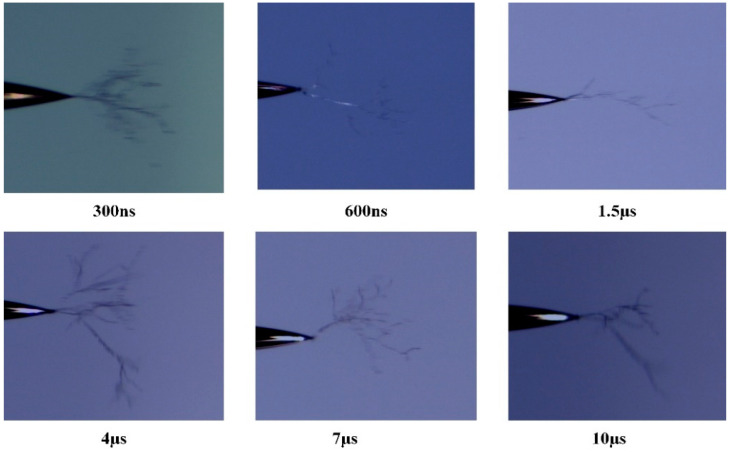
Morphology of electrical tree branches under pulse electric fields with different edge times.

The electrical treeing characteristics of silicone gel were tested as a function of the edge time of the pulsed electric field in Figure 4. The variation in the electrical treeing initiation voltage with the edge time of the square-wave pulse electric field is shown in Figure 5. The circles in the figure represent the average initiation voltage for electrical treeing at each edge time, and the error bars indicate the standard deviation of the initiation voltage. From the figure, it can be seen that the initiation voltage for electrical treeing decreases as the edge time increases. When the edge time is less than 1.5 µs, the slope of the curve is relatively steep, and the initiation voltage changes rapidly with the edge time. When the edge time exceeds 1.5 µs, the curve becomes flatter, and the initiation voltage for electrical treeing no longer changes significantly with the edge time. When the edge time increases from 300 ns to 1.5 µs, the initiation voltage for electrical treeing increases by 14.8%. However, when the edge time increases from 4 µs to 10 µs, the initiation voltage for electrical treeing only increases by 1.5%.

The experimental results are shown in Figure 5. When the edge time is 300 ns, the main trunk of the electrical tree is clearly visible, with more branches, a denser structure, and a larger fractal dimension. At 600 ns, the main trunk remains visible, but the number of branches slightly decreases. When the edge time increases to 1.5 µs, the number of branches significantly decreases. As the edge time increases to 4 µs, the electrical tree’s morphology becomes more complex, with the number of branches and the fractal dimension clearly increasing. When the edge time increases to 7 µs and 10 µs, the fractal dimension of the electrical tree shows a decreasing trend with increasing edge time.

A larger square-wave pulse electric field edge time corresponds to a higher electrical treeing initiation voltage, and a higher voltage amplitude causes greater material damage, making the electrical tree morphology more pronounced. By comparing the electrical tree morphology at edge times of 4 µs, 7 µs, and 10 µs, it can be observed that the initiation voltage for electrical treeing is almost the same under these three conditions. However, the morphology of the electrical tree becomes more complex as the edge time of the square-wave pulse electric field decreases. The experimental results indicate that, with similar electrical tree initiation voltages, the shorter the pulse electric field edge time, the more severe the material damage, and the more complex the electrical tree morphology.

### 2.3. Analysis of the Degradation Mechanism of Bubble Discharge Under a Pulsed Electric Field

The microscopic mechanism of bubble discharge was analyzed by quantitatively calculating the evolution of electron density, ion density, surface charge density, and electric field distribution.

Before the breakdown of the air gap, due to the lower externally applied voltage during this stage, the accumulation of space charge is not sufficient to exacerbate the distorted electric field that would lead to discharge in the air gap. During this stage, the smaller current present is the displacement current.

**Figure 6:**
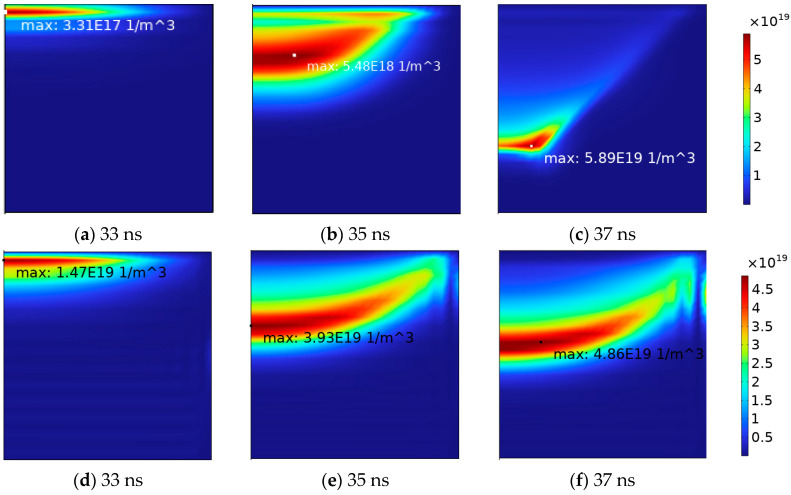
Spatial and temporal evolution of (**a**–**c**) electron density (**d**–**f**) and positive ion density during discharge.

**Figure 7:**
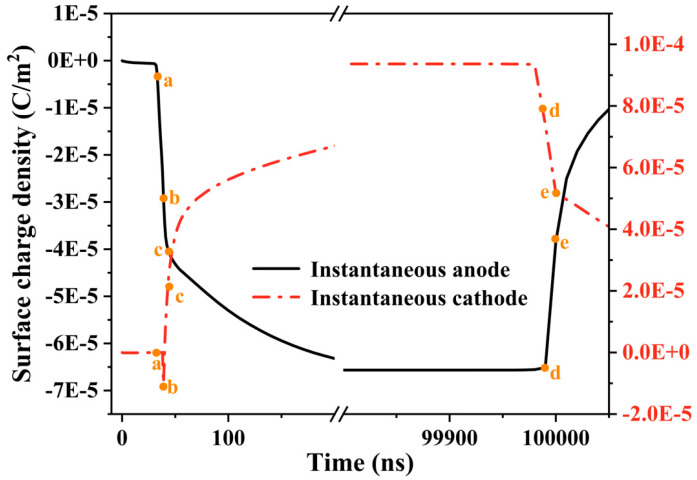
Evolution of surface charges.

As the gas voltage reaches a critical point, an increase in spatial charge causes distortion in the electric field, leading to a positive discharge event. Figure 6 illustrates the variations in electron and positive ion densities during the discharge process. Electron collapse is generated from the side of the high-voltage electrode, and generates a large number of positive and negative charges. Electrons and ions rapidly accumulate at the instantaneous anode and cathode, respectively, as depicted in Figure 7, which captures the evolution of space charge density. Starting from the breakdown, due to the higher mobility of electrons, the surface charge density at the interface of the instantaneous anode rapidly increases, leading to an extensive build-up of negative charge at the instantaneous anode.

**Figure 8:**
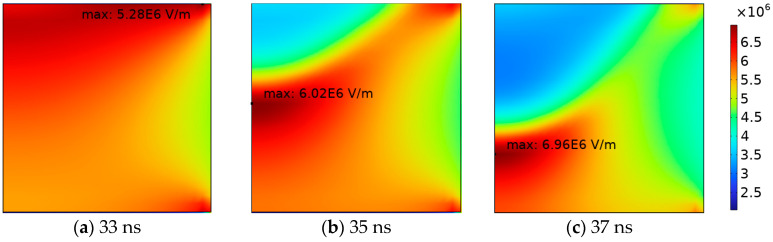
Spatial and temporal evolution of electrical field intensity during discharge.

During this phase, charges near the instantaneous cathode collide more violently and ionize. Under the influence of the electric field, electrons and positive ions continue moving toward the poles. The continuous increase in positive and negative charges, along with the differences in density and migration rates between them, results in the formation of a strong local electric field between the positive and negative charges. This significantly distorts the internal electric field of the gas. The spatiotemporal evolution of the electric field strength is illustrated in Figure 8.

It is precisely due to the significant difference in electron and ion migration rates under nanosecond pulse electric fields that they rapidly separate, exacerbating the distortion of the electric field and leading to the initiation of bubble discharge.

### 2.4. Analysis of the Mechanism of Material Degradation Affected by Discharge Products 

**Figure 9:**
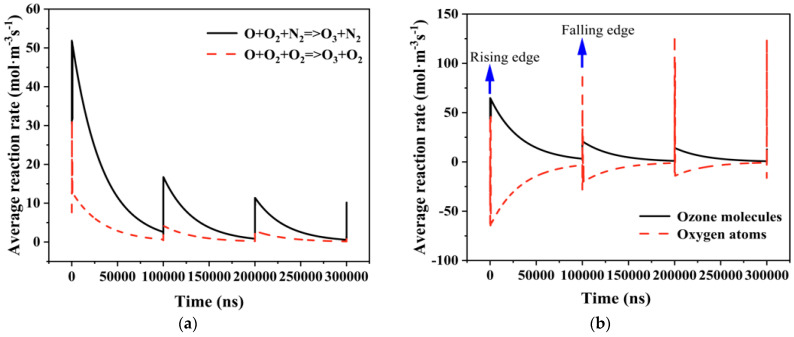
(**a**) Average reaction rate of oxygen atoms and ozone molecules in air gap over 1.5 cycles; (**b**) average chemical reaction rate of ozone generation.

This paper explores the mechanism of ozone-induced material destruction by examining the production and progression of ozone. Ozone molecules are mainly produced through three-body collision reactions: O + O_2_ + N_2_ → O_3_ + N_2_ and O + O_2_ + O_2_ → O_3_ + O_2_. Oxygen atoms are the primary source of ozone molecules. Figure 9a illustrates the average reaction rate of oxygen atoms and ozone molecules in the air gap at various times over 1.5 cycles. As seen in Figure 9b, the reaction rate between the two is higher at the pulse’s rising and falling edges. The average reaction rate of ozone molecules at the edges is positive, indicating an increase in the number of molecules, particularly at the first pulse’s rising edge, where the reaction rate is fastest, reaching up to 79 mol·m^−3^s^−1^. The average reaction rate of oxygen atoms is predominantly negative, signifying a decrease in atom count, except for at the pulse’s edge where it is positive. This suggests that oxygen atoms primarily form at the pulse’s edge. The creation of oxygen atoms is closely linked with electrons, and the sharp increase in electron numbers due to an electron avalanche at the pulse edge causes a significant rise in oxygen atoms generated by collision ionization. The conversion of oxygen atoms to ozone molecules also occurs intensely during these periods. In the area of the pulse voltage plateau, ionization reactions are less frequent, hence most oxygen atoms are slowly converted into ozone molecules. Figure 9a visualizes this process, showing two average chemical reaction rates for ozone generation, with a stronger reaction at the pulse’s edge.

**Figure 10:**
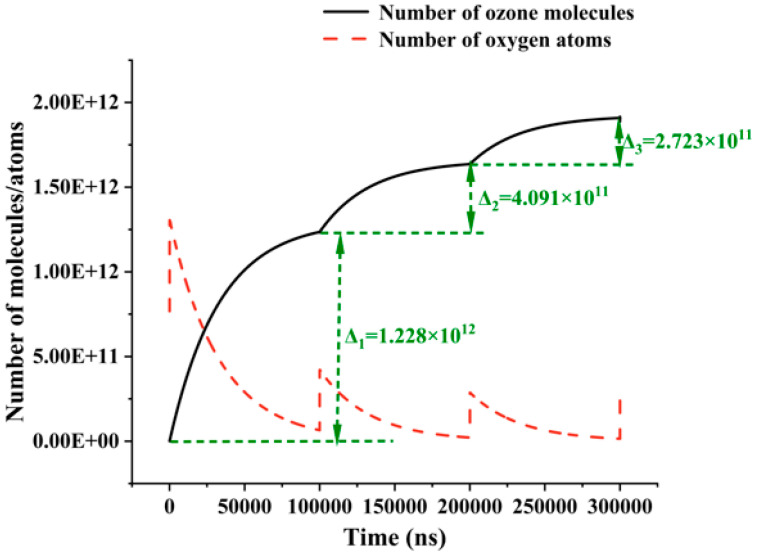
Variation in total number of oxygen atoms and ozone molecules in air gap with time.

The volume densities of oxygen atoms and ozone molecules were determined to plot the change in their respective quantities in the entire cylindrical air gap over time, as shown in Figure 10. As pressurization time increases, the number of oxygen atoms gradually decreases while the number of ozone molecules correspondingly increases. The small size of the air gap limits the number of available oxygen atoms inside it. Consequently, as the concentration of ozone molecules increases, the increment of ozone molecules generated by each half-cycle oxygen atom reaction gradually diminishes. In addition, the related literature indicates that after ultraviolet/ozone treatment or after a certain duration of corona discharge, a silica hardening layer forms on the siloxane surface, which may act as a diffusion barrier, gradually inhibiting the ozone generation rate [23].

Due to a concentration difference, the high concentration of ozone in the air gap leads to a gradual lateral and vertical diffusion, resulting in an even distribution of ozone molecules. Over time, with increasing pressurization, these ozone molecules distribute evenly within the air gap.

In summary, ozone molecules primarily originate near the electrodes at the pulse’s edge causing damage to the silicone gels. Compared to conventional DC electric fields, the unique ionization mechanism under pulsed electric fields results in a higher ozone generation efficiency, which intensifies charge accumulation and field distortion, leading to the deterioration of the material’s insulation properties.

### 2.5. Analysis of Bubble Discharge Mechanism Under Pulsed Electric Field

The influence of the operational conditions of a high-voltage semiconductor device on bubble discharge was explored using the aforementioned model parameters. By changing the pulse edge time and frequency, as well as the ambient temperature, we explored the evolution of discharge current and ozone molecules under differing operational conditions.

#### 2.5.1. Edge Time

**Figure 11:**
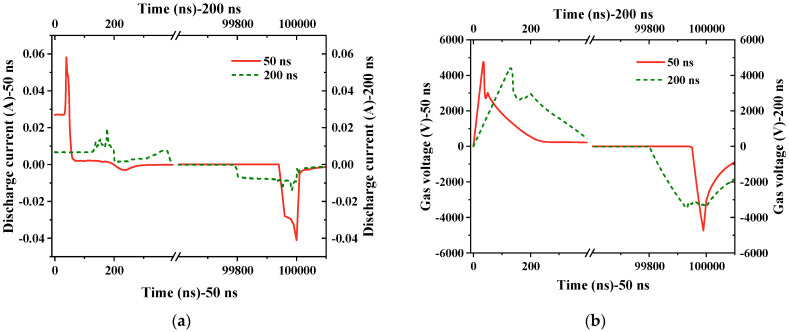
Discharge results within half a cycle for edge times of 50 ns and 200 ns. (**a**) Discharge current; (**b**) breakdown voltage.

First, with the edge time set to various values (50 ns, 200 ns, 400 ns, 1 μs, and 2 μs) and all other parameters held constant, the discharge plots for 50 ns and 200 ns were used as representative examples for analysis, as illustrated in Figure 11.

As shown in Figure 11a, an increase in pulse edge time results in a rise in the number of pulse discharge current peaks. Discharge occurs at the pulse edge or even within the pulse voltage plateau region, as each subsequent discharge peak is influenced by the residual charge from the first discharge peak. For this reason, the relationship between the first discharge current peak and the pulse edge is discussed here. It was found that the peak of the discharge current decreases rapidly as the pulse edge time increases. Discharge current is closely related to the gas voltage, and as shown in Figure 11b, and the pre-breakdown gas voltage increases along with the increase in the applied voltage change rate.

**Figure 12:**
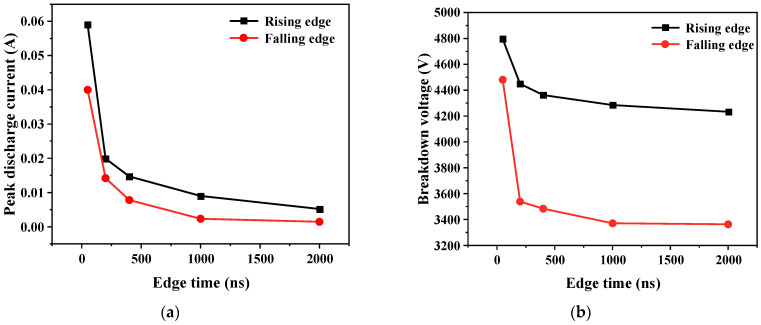
Variation in discharge current and breakdown voltage with pulse edge time. (**a**) Discharge current; (**b**) breakdown voltage.

The rate at which the gas voltage rises also accelerates with an increase in the applied voltage change rate, causing a relatively swift gas breakdown at a higher voltage level. Consequently, a higher gas voltage results in a larger discharge current. Therefore, as shown in Figure 12, as edge time decreases, both the breakdown voltage of the gas and the discharge current increase.

**Figure 13:**
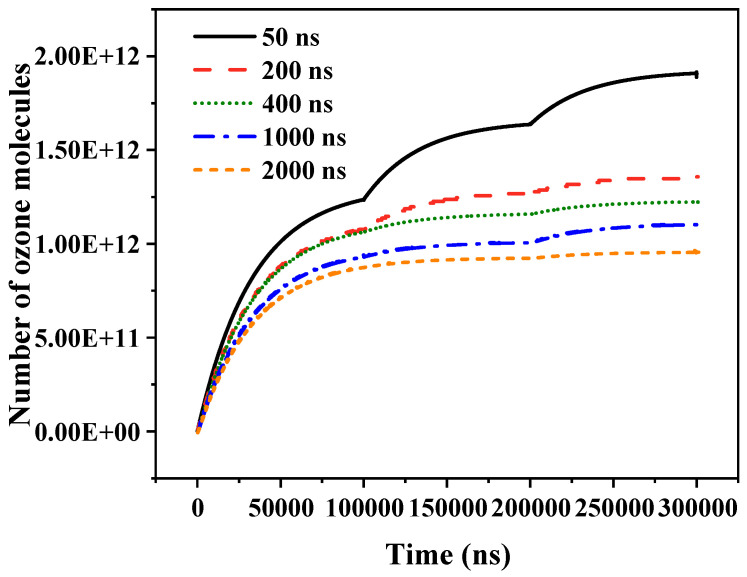
Variation in number of ozone molecules with pulse edge time.

Figure 13 illustrates the effect of edge time on ozone molecules in the air gap. A decrease in edge time resulted in an increase in the number of ozone molecules generated in the air gap over a specific period. From the above analysis of the ozone molecule generation process, it can be seen that the average reaction rate of the ozone molecules was higher at the pulse edge. The formation of ozone molecules was closely related to the discharge intensity. When the pulse edge time decreased, the discharge intensity at the pulse edge increased, leading to the production of a greater number of ozone molecules.

#### 2.5.2. Repetition Frequency

**Figure 14:**
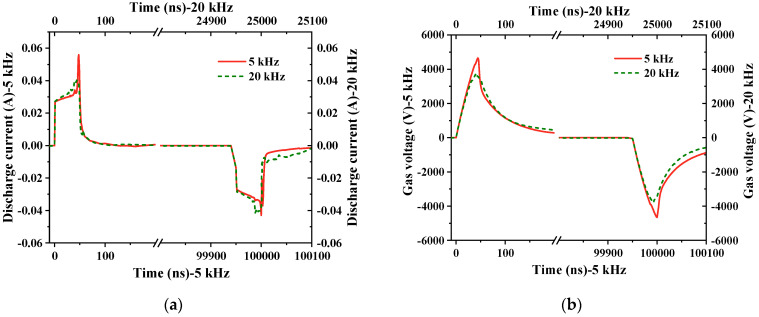
Results within half a cycle for repetition frequencies of 5 kHz and 20 kHz. (**a**) Discharge current; (**b**) breakdown voltage.

**Figure 15:**
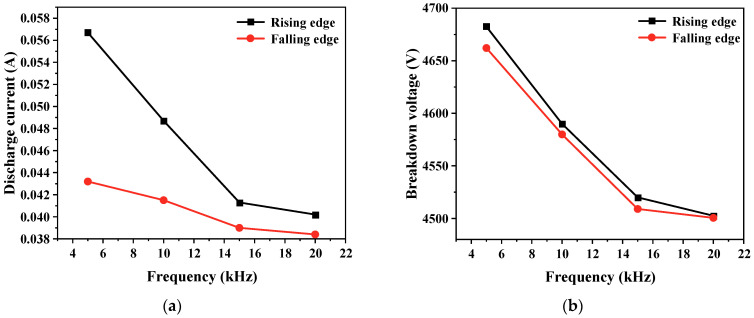
Variation in discharge current and breakdown voltage with pulse edge time. (**a**) Discharge current; (**b**) breakdown voltage.

The repetition rate of the pulse voltage is another critical factor impacting bubble discharge. Frequencies were set at 5 kHz, 10 kHz, 15 kHz, and 20 kHz, while other parameters remained constant, in order to explore the influence of pulse repetition rate on both discharge current and the number of ozone molecules over a certain time period. Figure 14 displays the discharge results over half a cycle at 5 kHz and 20 kHz repetition rates when the discharge is stable. With a constant edge time, the repetition rate affects the duration of the pulse plateau area. There is a memory effect during the discharge process where charged particles remaining in the electrode gap in the pulse plateau area influence the discharge at the pulse edge. As the frequency increases, the duration of both high- and low-level pulse states is reduced. The residual charge change in the discharge pulse at the edge of the previous pulse is less, leaving more electrons and positive ions in the discharge area. This has a greater impact on the discharge at the edge of the next pulse, resulting in the air gap breaking down earlier under a lower gas voltage. A smaller breakdown voltage, in turn, reduces the discharge current. The relationship between the discharge current, breakdown voltage, and pulse repetition rate is shown in Figure 15, with both the discharge current and breakdown voltage decreasing as the pulse repetition rate increases.

**Figure 16:**
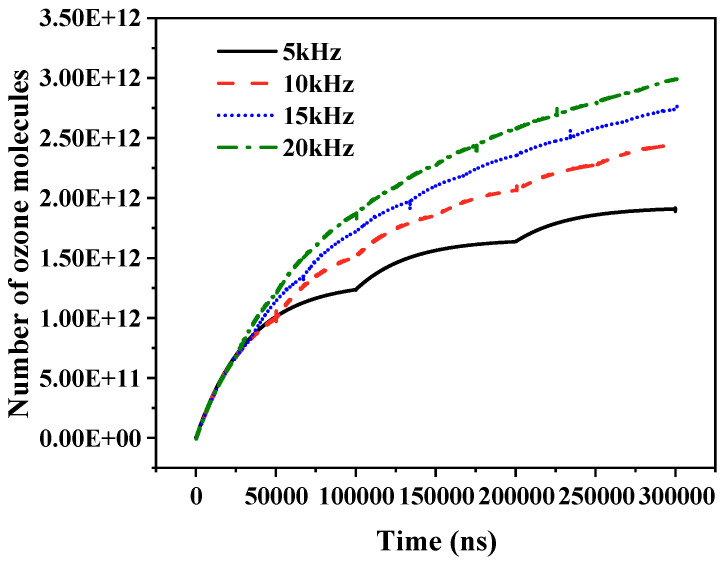
Variation in the number of ozone molecules with pulse repetition frequency.

Figure 16 shows the effect of repetition frequency on the number of ozone molecules over a specific time period. As the repetition frequency increases, the number of ozone molecules in the air gap increases over a given period of time. There are two reasons for this phenomenon: First, with an increased pulse period over a given time, bubble discharge at the edge occurs more frequently. The reaction rate, which is dominated by the ozone molecules produced by the edge discharge, also increases, leading to a higher production of ozone molecules. Second, due to the charge memory effect, the frequency increase causes the charge generated by the edge discharge to remain in the air gap. With more time to migrate or diffuse to the poles, a more frequent and robust reaction occurs in the air gap, thus producing more ozone molecules.

#### 2.5.3. Ambient Temperature

**Figure 17:**
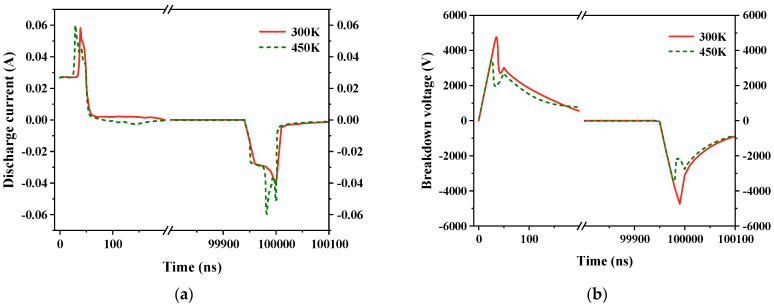
Discharge results in half a cycle for temperatures of 300 K and 450 K. (**a**) Discharge current; (**b**) breakdown voltage.

**Figure 18:**
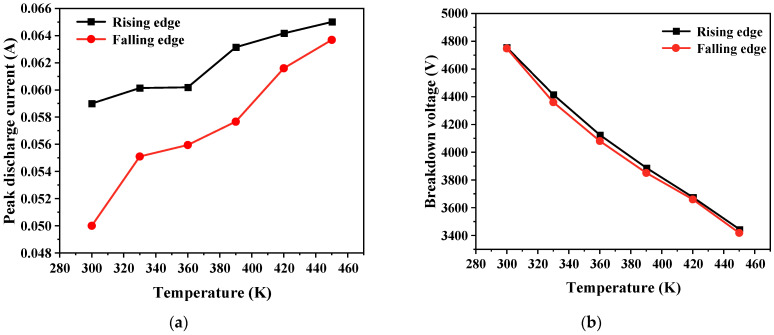
Variation in discharge current and breakdown voltage with temperature. (**a**) Discharge current; (**b**) breakdown voltage.

Ambient temperature is another significant factor affecting discharge. The ambient temperature was set at different levels: 300 K, 330 K, 360 K, 390 K, 420 K, and 450 K, respectively. This allowed us to ascertain the discharge current, breakdown voltage, and number of ozone molecules in the air gap at these different temperatures. Figure 17 displays the bubble discharge at 300 K and 450 K. When the temperature increases, bubbles break down earlier, resulting in a decrease in breakdown voltage, and an increase in discharge current. The peak of the discharge current also changes from one to two, indicating that multiple discharges occur at the pulse edge. This is because as the gas temperature increases, the distance between electrons and heavier particles enlarges, extending the free path of electrons. In the electric field, the electrons attain greater energy, collision ionization intensifies, and the quantity of charge rapidly increases. This leads to a decrease in the breakdown voltage, an increase in discharge current, and the occurrence of multiple discharges. The relationships among the discharge current, breakdown voltage, and temperature are shown in Figure 18.

**Figure 19:**
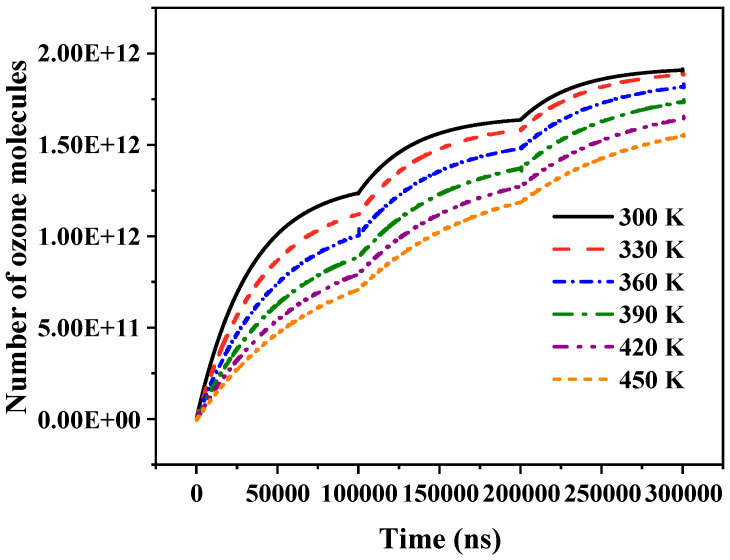
Variation in ozone molecule number with temperature.

An increase in temperature inhibits the formation of ozone molecules, as demonstrated in Figure 19. The number of ozone molecules diminishes with rising temperature. This is because the chemical properties of ozone are extremely unstable at high temperatures. The higher the temperature, the more violent the decomposition of ozone and the lower the ozone production rate; therefore, increasing temperature reduces the number of ozone molecules in the air gap over a certain time period.

In conclusion, different operating conditions promote or inhibit the discharge current and ozone molecules, thus influencing silicone gels insulation to varying degrees. A decrease in edge time enhances the discharge current and number of ozone molecules, thus promoting the deterioration of electric branches. An increase in repetition frequency promotes a reduction in breakdown voltage and an increase in ozone molecular number, in turn promoting deterioration. Similarly, an increase in temperature encourages a reduction in breakdown voltage and an increase in discharge current, in turn promoting deterioration.

## 3. Conclusions

In this study, a testing platform for electrical treeing in silicone gel under pulsed electric fields was established. The impact of voltage amplitude on the characteristics of bubbles within the silicone gel was investigated, and the relationship between bubbles and the formation of electrical treeing was explored. Additionally, the effects of different edge times and voltage amplitudes on the development of electrical treeing were tested. This article establishes a two-dimensional model of bubble discharge inside silicone gels under atmospheric pressure in a pulsed electric field. It quantitatively explores the spatiotemporal evolution of bubble discharge, discusses the characteristics of electric field distortion and ozone generation under the pulsed electric field, discusses the impact mechanism of bubble discharge on silicone gels dielectrics, and analyses the degree of damage to silicone gel insulation under the different operating conditions of high-voltage IGBTs, providing a theoretical and data-based foundation for the modification of silicone gels. The specific conclusions are as follows:

(1) When the square-wave pulse voltage amplitude is small, the bubbles are rapidly compressed due to material stress. In this experiment, when the voltage reaches 2 kV, unstable bubbles form near the needle tip. The electro-mechanical stress causes the bubbles to expand, but it is not sufficient to break through the constraints of the crosslinked network, so they do not develop into electrical treeing. However, when the pulse voltage amplitude exceeds a certain threshold, the bubbles near the needle tip rupture due to volume expansion, leading to the formation of electrical treeing. Experimental observations indicate that, in most cases, electrical treeing in silicone gel develops from bubbles. The rapid expansion of the bubbles under the excitation of the pulsed electric field, along with internal chemical processes, are the direct causes of the formation of electrical treeing.

(2) The edge time of pulsed electric fields has a significant impact on the electrical treeing characteristics of silicone gel. When the edge time is in the nanosecond range, the electrical tree initiation voltage increases sharply with the increase in edge time. However, when the edge time is in the microsecond range, the increase in electrical tree initiation voltage gradually decreases as the edge time increases. The transition region is roughly between 1 µs and 4 µs. Moreover, the electrical treeing morphology corresponding to different edge times of square-wave pulse electric fields also indicates that shorter edge times cause more severe damage to the silicone gel.

(3) Bubble discharge is prone to occur at the edges of pulses due to the rapidly changing electric field and the electron migration rate being much higher than that of positive ions. The separation of positive ions and electrons will exacerbate the internal electric field distortion within the bubble, triggering and promoting electron avalanches, eventually leading to streamer discharge. The model validates the unique charge response mechanism under pulsed electric fields and quantitatively calculates the degree of electric field distortion.

(4) Ozone molecules increase sharply at the cathode, while in the flat region of the pulsed voltage, the growth rate of ozone molecules slows down, leading to the diffusion of ozone molecules from the cathode to the surrounding areas. This is primarily because intense ionization collisions tend to occur at the pulse edge in the pulsed electric field. Here, the concentration of oxygen atoms is high, and the reaction to generate ozone is vigorous. However, as time increases, the rate of ozone production significantly decreases, and the concentration of ozone gradually reaches a saturation point. The model quantitatively analyzes the mechanism of ozone formation under pulsed electric fields.

(5) Shortening the pulse edge time increases the breakdown voltage, discharge current, and the number of ozone molecules. In this case, both the discharge current and ozone molecules become the main factors contributing to material deterioration. An increase in repetition frequency decreases both the breakdown voltage and discharge current, yet it increases the number of ozone molecules over a certain period. Here, ozone molecules act as the main factor causing material deterioration. Similarly, an increase in temperature results in a decrease in breakdown voltage and an increase in discharge current. However, higher temperatures decrease the number of ozone molecules in the air gap. In this scenario, the discharge current becomes the main factor causing material deterioration. This model provides a quantitative representation of how the different operating conditions of a high-voltage IGBT can affect the deterioration mechanism of silicone gels.

The results of this study are of theoretical significance to further understanding the bubble discharge mechanism of silicone gels under a pulsed electric field and revealing the mechanism of the deterioration of silicone gels under the different operating conditions of a high-voltage IGBT. This study will provide data support for future advances in silicone gels.

## 4. Materials and Methods

### 4.1. Experimental Section

#### 4.1.1. Insulation Material

**Figure 20:**
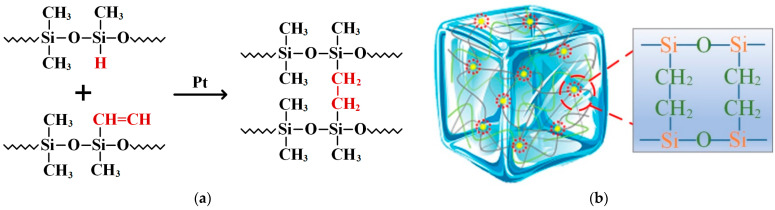
(**a**) Chemical equation for silicone hydrogenation reaction. (**b**) Silicone gel crosslinking system.

Based on a comprehensive review of references, WACKER’s addition-curing silicone gel was selected for this study. Component A is a hydrogen-containing silicone oil, and Component B is Vinylmethylsiloxane homopolymer [3,4,5,20,21,22]. Both components A and B are colorless, odorless, oily liquids. The hydrogen-containing silicone oil and Vinylmethylsiloxane crosslink under the action of a catalyst, undergoing a hydrosilylation reaction under suitable conditions. The vinyl content was controlled at 2–5% relative to the total molecular weight. The platinum-based catalyst was used at a concentration of approximately 10–30 ppm relative to the total mixture weight. Since the hydrosilylation reaction triggered by the platinum catalyst proceeds relatively quickly at room temperature, about 0.01–0.1% of Inhibitor PT 88, a propargyl alcohol-type compound, was used to delay the reaction initiation, ensuring sufficient time for the mixing and degassing steps. The chemical reaction equation is shown in Figure 20a. The mixture eventually cures into a transparent, viscoelastic gel, forming a polymeric solid network. Any unreacted liquid portion is evenly distributed in the pores between the crosslinked structures, with its main component being polydimethylsiloxane (PDMS) and its derivatives, as shown in Figure 20b. The product has a viscosity of 1000 mPa·s, a dielectric strength of 23 kV/mm, a volume resistivity of 10¹⁶ Ω·cm, and a dielectric constant of 2.7.

The preparation process of the silicone gel sample was as follows: First, components A and B were mixed at a 1:1 mass ratio. The mixed sample was then placed in a three-dimensional high-speed mixer and dynamically rotated at 2500 r/min for 60 s. After mixing, the sample was allowed to rest for 1 min, and this step was repeated five times. The mixed sample was then degassed: the sample was injected into a mold and degassed under a vacuum of −100 kPa for 30 min. Finally, the mold was placed in a vacuum drying oven and cured at 100 °C for 1 h to obtain the gel-like sample.

#### 4.1.2. Electric Tree Branch Testing Platform Under Electric Thermal Coupling Field

**Figure 21:**
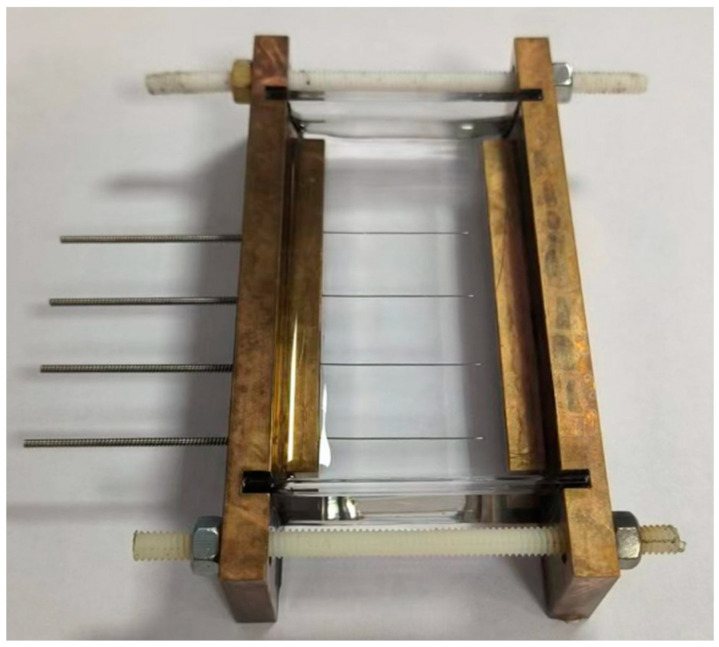
Silicone gel electric tree mold.

The physical image of the electrical treeing mold is shown in Figure 21. It consists of a high-voltage electrode, a grounded electrode, needle electrodes, and a silicone gel sample. Small holes are drilled on the high-voltage electrode, with four stainless steel needles arranged in sequence at a 12 mm spacing. The radius of curvature at the needle tips is 3.4 μm. During testing, the voltage is directly applied to the high-voltage electrode in contact with the stainless steel needles, while the electrode on the other side is directly grounded. According to relevant literature, when the distance between the needle tips exceeds 10 mm, the electric fields generated by adjacent needle tips do not interfere with each other, allowing the electrical treeing characteristics at the tips of all four needles to be observed simultaneously [24].

**Figure 22:**
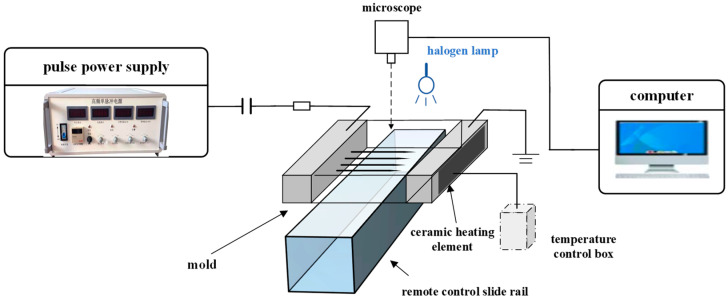
Silicone gel electrical treeing test platform under pulsed electric field.

The electrical treeing testing platform under the thermo-electrical coupling field is shown in Figure 22. It consists of a pulsed power supply, connection circuits, electrical treeing molds, remote control tracks, halogen lamps, a microscope, a temperature control system, and a computer. The pulsed power supply is the HVP-10B model produced by Xi’an Lingfeng Yuan Electronics Technology Co., Ltd. (Xi’an, Shaanxi, China), which can output a negative polarity square-wave pulse voltage with an adjustable amplitude from 0 to 10 kV, an adjustable frequency from 0 to 10 kHz, an edge time of 50 ns, and a duty cycle of 50%.

The temperature of the mold is controlled by the temperature control box and ceramic heating plates. The sensor in the temperature control box measures the temperature near the needle tip, and the feedback signal is used to adjust the heating power of the heating plates, ensuring that the temperature of the tested sample remains at the preset value. Since the sample is subjected to a high-amplitude electric field, and to prevent breakdown of the temperature control system, both the heating plates and the temperature sensors are made from high-voltage insulating ceramics, ensuring that the temperature control system is not affected by the high-voltage circuit.

The microscope used was the Motic SMZ-171TL optical microscope (Motic Incorporation Ltd., Barcelona, Spain), with an adjustable magnification range of 0 to 50 times. During testing, the microscope was positioned above the needle tip, and image signals were transmitted to a computer via a Charge-Coupled Device (CCD) camera, allowing observation and recording of the electrical treeing characteristics at the needle tip. To ensure proper brightness in the field of view, a halogen lamp with a cold light source was used to illuminate the sample, providing additional light while preventing temperature rise due to overheating. The electrical treeing mold was fixed to a remote-controlled track, which can be adjusted by remote control, allowing the four needles to be sequentially exposed within the microscope’s field of view. This enabled observation of the electrical treeing characteristics at different needle tips.

#### 4.1.3. Experimental Methods

The bubble characteristics of the silicone gel were tested using the testing platform described in Section 4.1.2. A negative polarity square-wave pulse electric field with a frequency of 1000 Hz was applied to the sample. A stepwise voltage increment method was used, starting with an initial voltage amplitude of 2 kV. The pulsed electric field was continuously applied to the sample, with the voltage amplitude slowly increasing until electrical treeing occurred at the needle tip. To clarify the relationship between the bubble morphology changes at the needle tip and the initiation of electrical treeing, a Charge-Coupled Device (CCD) camera was used to accurately record the dynamic process of electrical treeing formation at the needle tip in real time, with a frame rate of 24 frames per second. The video was then processed frame by frame using video editing software to capture the process of electrical treeing formation.

The variation in the electrical treeing characteristics of silicone gel with respect to edge time was tested. A stepwise voltage increment method was used, starting with an initial voltage amplitude of 2 kV applied to the needle electrode. If no electrical treeing was observed within 5 min, the voltage amplitude was increased by 0.2 kV, and the process was repeated until electrical treeing occurred at the needle tip. The edge times of the square-wave pulse electric field were set to 300 ns, 600 ns, 1.5 µs, 4 µs, 7 µs, and 10 µs, in order to investigate how the initiation voltage and morphology of electrical treeing change with the edge time of the square-wave pulse electric field.

### 4.2. Simulation of Bubble Discharge in Silicone Gel

#### 4.2.1. Geometry and Initial Conditions

**Figure 23:**
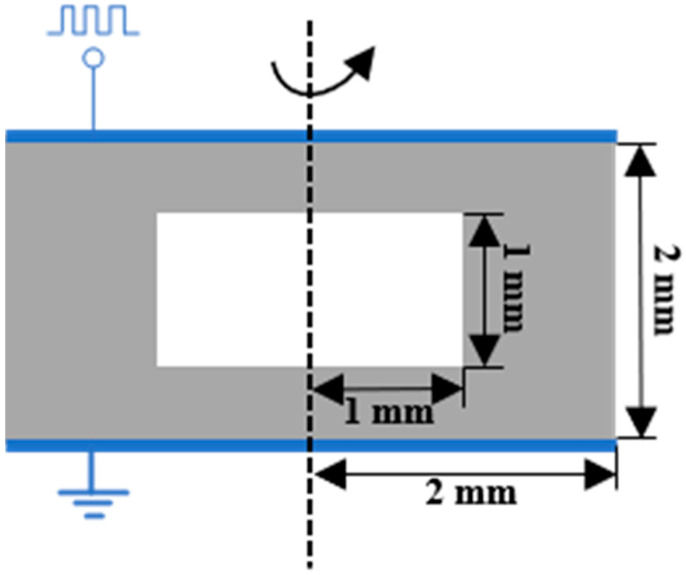
Geometry of simulation model.

This paper is based on the Plasma Module of COMSOL Multiphysics. The insulation material selected is the same as the experimental material, which is WACKER’s addition-curing silicone gel. The model’s geometry is depicted in Figure 23. To enhance the efficiency and convergence of the calculation, the bubble model within the solid medium was simplified. A two-dimensional axisymmetric model was used to simulate the cylindrical solid medium encapsulating an air gap. The insulating medium’s total thickness was 2 mm with a radius of 2 mm, while the internal air gap’s thickness was 1 mm with a radius of 1 mm. Given that the relative permittivity of silicone gels ranges from about 2.5 to 3.5, the relative permittivity of the insulating medium was set at 2.7. The air gap was filled with air at atmospheric pressure and a temperature of 300 K. A unipolar pulse voltage with an amplitude of 10 kV, a frequency of 5000 Hz, rising and falling edge times of 50 ns, and a duty cycle of 50% was applied to the top layer of the insulating medium.

In the study, to reduce the computational load, this model does not consider N^+^ and N_3_^+^ ions, as they quickly convert to N_2_^+^ [25]. Additionally, since the concentrations of O^+^, O_3_^−^, and O_4_^−^ are relatively low, they are not included in the reactions. Moreover, because nitrogen oxides account for only 0.2% of the mixture, their significance is less than that of ozone, which makes up 2%, and thus reactions related to nitrogen oxides are not considered [26]. The photoionization module was excluded within the parameter range considered in this study. The main reason is that the small air gap limits the electron heating rate and the recombination rate of positive and negative ions, resulting in negligible photoionization [27]. The initial electron density was set to 1 × 10^17^ m^−3^. To maintain the electrical neutrality of the original gas, the N_2_^+^ particles were set to an initial value derived from the neutrality constraint [28]. Taking into account the composition of air, N_2_ was set based on mass constraint, the initial molar fraction of O_2_ was set to 0.21, and the initial molar fractions of all other gasses were set at 1 × 10^−16^. The parameters ensure the simulated environment closely approximates real atmospheric conditions, allowing for accurate modeling of gas behavior and interactions under the influence of a pulsed electric field.

#### 4.2.2. Model Control Equations and Boundary Conditions

The model simulates charge transport using a drift–diffusion equation, wherein the control equations for electron density and electron energy density are as follows:

(1) $$\frac{\partial }{\partial t}(n_{e})+\nabla \cdot \Gamma_{e}=R_{e}-(u\cdot \nabla )n_{e},$$

(2) $$\Gamma_{e}=-(\mu_{e}\cdot E)n_{e}-\nabla (D_{e}n_{e}),$$

(3) $$\frac{\partial }{\partial t}(n_{\varepsilon })+\nabla \cdot \Gamma_{\varepsilon }=R_{\varepsilon }-(u\cdot \nabla )n_{\varepsilon },$$

(4) $$\Gamma_{\varepsilon }=-(\mu_{\varepsilon }\cdot E)n_{\varepsilon }-\nabla (D_{\varepsilon }n_{\varepsilon }),$$

where *n_e_* and *n_ε_* represent electron density and electron energy density, respectively; *Γ_e_* and *Γ_ε_* represent electron fluxes and electron energy fluxes, respectively. Re denotes the electron density source term, and *R_ε_* signifies the electron energy density source term. The variable u signifies fluid velocity, whereas *μ_e_* and *μ_ε_* represent the mobility of the electron and electron energy density, respectively, with the latter characterized by the mobility of the electron.

The transport of heavy substances, including neutral particles and ions, can be described using the mixture average diffusion equation as follows:

(5) $$\rho \frac{\partial }{\partial t}(\omega_{k})+\rho (u\cdot \nabla )\omega_{k}=\nabla \cdot j_{k}+R_{k},$$

where *ρ* represents the density of the heavy matter mixture, *ω_k_* denotes the mass fraction of the kth heavy substance solved, *j_k_* signifies the mass flux of the kth heavy matter, and *R_k_* represents the mass source term of the kth heavy matter.

All types of heavy substances must adhere to mass conservation conditions, and the density, *ρ*, of the heavy matter mixture is calculated based on an ideal gas.

As the density of heavy matter ions is proportional to their mass fraction, the continuity equation of heavy matter can be derived from the average diffusion equation of heavy matter:

(6) $$\frac{\partial }{\partial t}n_{k}+\nabla \cdot \Gamma_{k}=s_{k},$$

where *n_k_* denotes the density of the *k*th heavy matter, *Γ_k_* is the flux, and *s_k_* represents the source term. The potential distribution during discharge can be described by the Poisson equation as below:

(7) $$-\nabla \cdot \varepsilon_{0}\varepsilon_{r}\nabla V=\rho ,$$

where *ε*_0_ represents the permittivity of a vacuum, *ε_r_* is the relative permittivity, and the charge density ρ can be calculated using the number density of electrons and other heavy ions:

(8) $$\rho =e(\sum_{k=1}^{n}z_{k}n_{k}-n_{e}),$$

the expression for the accumulation of charged particles on the surface of the boundary medium and the effect of the surface charge on the electric field is given below:

(9) $$\frac{d\sigma_{s}}{dt}=n\cdot e(\Gamma_{p}-\Gamma_{e}),$$

(10) $$\sigma_{s}=n\cdot (D_{1}-D_{2}),$$

where *σ_s_* represents the surface charge density, while *D*_1_ and *D*_2_ denote the electrical displacements in the gas and dielectric plates, respectively.

#### 4.2.3. Chemical Reaction Equation

To facilitate calculations, a simplified chemical reaction model is adopted, primarily focusing on the nitrogen–oxygen reaction system. This includes 25 reactions and 11 substances, encompassing electrons, positive ions (N_2_^+^, O_2_^+^, O_4_^+^, N_4_^+^), negative ions (O^−^, O_2_^−^), ground state atoms (N, O), and ground state molecules (N_2_, O_2_, O_3_). The detailed gas phase reactions are presented in Table 1 and Table 2.

In these tables, N_A_ is Avogadro’s constant, and $f_{\left(\bar{\varepsilon }\right)}$ is the electron collision reaction rate coefficient, which is calculated by entering the cross-section coefficient into the Bolsig+ software (version 03/2016) [29]. The calculation formula is given below:

(11) $$f(\bar{\varepsilon })=\int_{0}^{\infty }\sqrt{\frac{2e}{m_{e}}}\varepsilon \sigma_{l}F_{\bar{\varepsilon }}(\varepsilon )d\varepsilon ,$$

where *σ*_l_ represents the collision cross-section of the reaction, $F_{\bar{\varepsilon }}$ denotes the electron energy distribution function, and *m_e_* represents the electron mass.

The plasma region is discretized using quadrilateral grids, whereas the dielectric domain employs triangular grids. In this study, a combination of square and triangular meshes is chosen. At this point, the model consists of 44,400 elements and 880 boundary elements, with the largest element measuring 0.006 mm and the smallest at 0.00001 mm. Continuing to refine the mesh does not significantly alter the simulation outcomes but greatly increases the simulation time.

For the solver settings, output time steps are interpolated. At pulse edges, the time step is set to 1 nanosecond, and in non-edge areas, it is set to 10 nanoseconds. The solver computation time step uses the system’s default precision mode, with an initial step size set to 1 × 10^−13^, and the maximum step size constraint set to automatic, allowing the solver to adjust the step size constraint automatically as needed during the calculation.

**Table 1:** Plasma Chemistry of Air Discharge.

Serial Number	Reactive	Rate Constant	Reaction Energy/eV	References
1	e + N_2_ → 2e + N_2_^+^	$f(\bar{\varepsilon })\;(m^{3}s^{-1})$	15.6	[30]
2	e + O_2_ → 2e + O_2_^+^	$f(\bar{\varepsilon })\;(m^{3}s^{-1})$	12.06	[31]
3	e + O_2_ → e + 2O	$f(\bar{\varepsilon })\;(m^{3}s^{-1})$	5.58	[31]
4	e + O_2_ → O_2_^−^	$f(\bar{\varepsilon })\;(m^{3}s^{-1})$	/	[32]
5	e + O_2_ → O + O^−^	$f(\bar{\varepsilon })\;(m^{3}s^{-1})$	/	[32]
6	e + O_4_^+^ → 2O_2_	$2.5\times 10^{-13}T_{e}^{-0.5}\;(m^{3}s^{-1})$	/	[33]
7	e + O_3_^−^ → O + O_2_^−^	$1\times 10^{-15}\;(m^{3}s^{-1})$	/	[34]
8	e + O_2_^+^ → 2O	$1.2\times 10^{-14}T_{e}^{-0.7}\;(m^{3}s^{-1})$	/	[33]
9	e + 2O_2_ → O_2_ + O_2_^−^	$5.65\times 10^{-43}T_{e}^{-3}\;(m^{6}s^{-1})$	/	[33]
10	e + N_2_^+^ → N + N	$4.8\times 10^{-13}{\left(T_{e}/0.026\right)}^{-0.5}\;(m^{3}s^{-1})$	/	[33]
11	e + N_2_^+^ + N_2_ → 2N_2_	$6.07\times 10^{-34}{T_{e}}^{-3}\;(m^{6}s^{-1})$	/	[29]
12	e + O + O_2_ → O + O_2_^−^	$f(\bar{\varepsilon })\;(m^{3}s^{-1})$	/	[29]
13	e + O + O_2_ →O_2_ + O^−^	$f(\bar{\varepsilon })\;(m^{3}s^{-1})$	/	[32]
14	O_2_ + N_2_^+^ +N_2_ → N_4_^+^ + O_2_	$5\times 10^{-41}\;(m^{6}s^{-1})$	/	[35]
15	O_2_ + N_4_^+^ → 2N_2_ + O_2_^+^	$2.5\times 10^{-16}\;(m^{3}s^{-1})$	/	[35]
16	O_2_^−^ + O_4_^+^ + O_2_^−^ → 4O_2_	$2\times 10^{-37}\;(m^{6}s^{-1})$	/	[29]
17	O_2_^−^ + O_4_^+^ + N_2_^−^ → 3O_2_ + N_2_	$2\times 10^{-37}\;(m^{6}s^{-1})$	/	[29]
18	O_2_^+^ + 2O_2_^−^ → O_4_^+^ + O_2_	$2.4\times 10^{-42}\;(m^{6}s^{-1})$	/	[35]
19	N_2_^+^ + O_2_^−^ → N_2_ + O_2_^+^	$2\times 10^{-12}\;(m^{3}s^{-1})$	/	[35]
20	O^−^ + O_2_^+^ → O + O_2_-	$1\times 10^{-13}\;(m^{3}s^{-1})$	/	[36]
21	O_2_ + O_4_^+^ → 3O_2_	$2\times 10^{-37}\;(m^{6}s^{-1})$	/	[35]
22	O_2_^−^ + O_2_^+^ + N_2_ → 2O_2_ + N_2_	$1.19\times 10^{-39}\;(m^{6}s^{-1})$	/	[36]
23	O_2_^−^ + O_2_^+^ + O_2_ → 3O_2_	$5.54\times 10^{-40}\;(m^{6}s^{-1})$	/	[36]
24	O + O_2_ + O_2_ → O_3_ + O_2_	$6.2\times 10^{-46}\;(m^{6}s^{-1})$	/	[35]
25	O + O_2_ + N_2_ → O_3_ + N_2_	$6.9\times 10^{-46}\;(m^{6}s^{-1})$	/	[35]

**Table 2:** Surface Plasma Chemistry of Air Discharge [33].

Serial Number	Reactive	Secondary Emission Coefficient	Secondary Electron Average Energy/eV
1	N_2_^+^ + Surface → N_2_	$6.5\times 10^{-4}\;(m^{6}s^{-1})$	3
2	O_2_^+^ + Surface → O_2_	$6.5\times 10^{-4}\;(m^{6}s^{-1})$	3
3	N_4_^+^ + Surface → 2N_2_	$6.5\times 10^{-4}\;(m^{6}s^{-1})$	3
4	O_4_^+^ + Surface → 2O_2_	$6.5\times 10^{-4}\;(m^{6}s^{-1})$	3
5	O_2_^−^ + Surface → O_2_	0	0
6	2O + Surface → O_2_	0	0

## Data Availability

Data are contained within the article.
